# Compassionate mind training as school-based social and emotional learning: extension to younger pupils and effects of programme dosage

**DOI:** 10.3389/fpsyg.2026.1861788

**Published:** 2026-09-22

**Authors:** Frances A. Maratos, Fabio Parente, Julie Hurst, Caroline Harvey

**Affiliations:** Holistic Health and Wellbeing Group, Department of Psychology, College of Science and Engineering, University of Derby, Derby, United Kingdom

**Keywords:** anxiety, education, emotion regulation, iatrogenic harm, prevention, school, SDG#3, wellbeing

## Abstract

**Introduction:**

The transition from primary/elementary to high-school education is associated with increased mental health difficulties, especially anxiety. Previously, compassionate mind training (CMT) has been demonstrated to stem anxiety increases and improve prosocial behaviours in children aged 11–12. We extended this research by exploring CMT training effects via two further studies: (i) younger children aged 10–11, and (ii) a shortened/compacted delivery length with children aged 11–12.

**Method:**

We used a mixed methods convergent parallel approach, collecting both quantitative and qualitative data from 110 pupils (*m* = 56; female = 52; 2 = other) using randomised cluster-controlled designs. In Study 1 (S1), 60 primary pupils aged 10–11 took part in standard Personal, Social, Health and Economic (PSHE) lessons (control *n* = 30) or CMT lessons (experimental *n* = 30) over a 6-week period. In Study 2 (S2), 50 secondary pupils aged 11–12 took part in standard PSHE (control *n* = 25) or CMT (experimental *n* = 25) lessons over a 4-week period. Data collected included lesson implementation efficacy, standardized measures of anxiety, perfectionism, compassion, stress, wellbeing, and open-ended question data.

**Results:**

S1 Pupils reported positively on their experiences of the CMT lessons, content, and practices. Mixed-factorial ANOVAs demonstrated reductions in trait anxiety and increases in self-compassion and general wellbeing for the CMT group only. For the CMT lessons, thematic analyses further revealed that content and practices positively impacted emotion understanding and literacy, emotion regulation, and prosociality. S2 Implementation efficacy was considerably lower than in the previous research and S1. Mixed-factorial ANOVAs returned no significant interaction effects. Thematic analyses revealed that lesson content and practices positively impacted emotion understanding, regulation, and the classroom climate, but also highlighted challenges of compacted delivery.

**Discussion:**

This research demonstrated repeated efficacy of the CMT lessons with younger children, but reduced self-wellbeing effectiveness with same-age children when the number of delivery sessions was reduced. We argue that this is because social and emotional learning (SEL) requires time to embed, including repeated practice of the introduced knowledge, practices, and skills. Additionally, as a policy response to ongoing PSHE/SEL concerns in the UK, our research demonstrates an example of best practice methodology, balancing power, rigour, and mixed methods designs with ethical and moral requirements to minimise the potential for harm.

## 1 Introduction

Globally, the number of children and young people experiencing mental health difficulties is rising (Newlove-Delgado et al., 2023). To counter this, policymakers, clinicians, and academics have supported and promoted wellbeing approaches and initiatives in school settings, given that children and adolescents spend many of their waking hours in school. Preventative strategies within schools, such as universal approaches designed for all pupils, can be particularly effective in supporting child mental health and wellbeing (National Governance Association, 2024; Cary and Webb, 2025). Thus, a current focus of many governments is access to effective mental health and wellbeing support in schools (e.g., Department for Education, UK, 2021; Ministry of Health, New Zealand, 2021; Public Health Agency of Canada, 2021). However, concerns have been raised regarding the effectiveness of such interventions, including the potential for them to cause harm (Foulkes and Stringaris, 2023). This could reflect that several initiatives lack theoretical grounding, are of poor-quality study design, and/or have undergone limited or no proper trial evaluation (Kuyken et al., 2023; Cary and Webb, 2025).

Recently, Maratos et al. (2024) demonstrated that a suite of lessons based on emotion regulation and compassionate mind training (CMT) literature and practices could be a promising school-based wellbeing social and emotional learning (SEL) curriculum for improving prosocial behaviours, enhancing the classroom environment, and protecting against deteriorations in child mental health. In a small-scale mixed-methods randomised cluster-control trial, Maratos et al. (2024) demonstrated that the lessons were not only engaging (as rated by the pupils involved in the trial) but were also effective in thwarting increases in anxiety and improving emotion regulation, kindness to others, and feelings of inclusion. The proposed mechanism of action was CMT. CMT is a theoretically informed biopsychosocial approach to behaviour (Gilbert, 2025; Gilbert and Simos, 2022; Maratos and Harvey, 2024), with meta-analyses in adult populations revealing medium-to-large effect sizes for reducing overall negative mental health outcomes and improving compassion for oneself and others (Petrocchi et al., 2024).

Maratos et al. (2024) designed the lessons for children transitioning from primary (elementary) to secondary (high) school, as the transition from primary to secondary school education (Year 7) is one of the most stressful events in a child’s life (Zeedyk et al., 2003). For example, in the UK, a recent survey reported that 28% of secondary school children avoided school due to anxiety (Campbell, 2024). As a next step, Maratos et al. (2024) suggested the exploration of CMT-Pupils across wider demographics, including those who are just approaching the primary/elementary school transition. This is because the transition from childhood to adolescence, particularly in the 9–11-year age bracket, appears to coincide with major physical, psychological, and social changes (Sawyer et al., 2018). Thus, early intervention may be key to stemming the observed increases in the prevalence of mental health difficulties from childhood to adolescence (Vizard et al., 2020). In the UK specifically, the children’s commissioner for health reported that in September 2024, NHS statistics revealed over 500 under 17-year-olds per day in England were referred to mental health services for anxiety (DeSouza, 2024). This is more than double the rate before the Covid-19 pandemic. With over 40,000 children experiencing NHS waitlists of over 2 years for specialist support, de Souza argued that early preventative strategies are essential, given that current demand for specialist services is massively outstripping resources. Relatedly, Gutman and Vorhaus (2012) argued that children with better emotional wellbeing in primary school make better progress in their current school and, subsequently, are more engaged in secondary school, leading to a multitude of wellbeing benefits. By age 13, these include greater school enjoyment, as well as emotional, behavioural, social, and school wellbeing.

Additionally, Perkins et al. (2022), in their review of compassion and kindness school-based interventions, found that compassion and prosocial interventions were more effective with younger age groups. Two reasons for this finding may include that: (i) children aged 10–11 have a good mastery of first-level theory of mind, which is needed for perspective-taking, such as understanding the emotional state of another (Korkmaz, 2011); and (ii) children aged between 7 and 11 years demonstrate increasing capacities for perspective-taking and empathic concern, enabling greater moral concern as well as moral concern expansiveness (Neldner et al., 2023). Moral concern is the sense of obligation to protect, invest in, or defend another entity’s wellbeing. Expansiveness is the extent to which this moral concern is applied to others. However, recent research has revealed that children aged 10 can begin to demonstrate a narrowing of their moral concern expansiveness (or circle) to only those within close relationships, particularly in Western cultures (Marshall et al., 2025). Therefore, introducing children aged 10–11 to the concept of compassion and its three flows may be critical in limiting this narrowing of expansiveness, as children of this age can internalise moral standards while remaining malleable to outside influence. In sum, there is good evidence that for children aged 10–11, compassion-based social and emotional learning may protect against mental health deterioration, promote wellbeing, and foster positive virtue formation.

A further factor of importance when considering the impact of SEL is intervention (or programme) dosage. Intervention dosage is a term used to refer to various aspects of an intervention, such as its length as defined by the number of sessions, total time, or frequency of sessions. In several recent reviews and meta-analyses, while Perkins et al. (2022) found no evidence that ‘intervention dosage’—that is, the length of the SEL—affects intervention effectiveness, Durlak et al. (2022) found that shorter programme durations predicted better outcomes than longer programmes. Similarly, Cohen et al. (2024) found that interventions consisting of ≤4 sessions (or ≤240 min of intervention time) still led to significant improvements in mental health and wellbeing compared to control conditions, whereas Lemberger‐Truelove et al. (2025) found no effects of SEL intervention frequency (defined by the number of sessions) on effect sizes. Some evidence, however, suggests that what is key to SEL programmes is the time taken to embed learning, including repeated introduction and practice of the knowledge, practices, and skills introduced, especially where SEL is concerned (e.g., Weare, 2015). In specific reference to CMT, Healy et al. (2025) found no benefits of brief compassion training for school employees above those of a standard focused professional development workshop. This result aligns well with the argument that emotion regulation and compassionate self-responding are skills that typically require repeated practice, ongoing reflection, and opportunities to revisit and consolidate learning before stable changes are observed (Gilbert and Procter, 2006; Maratos et al., 2019; Matos et al., 2022; Cefai et al., 2022). It is therefore important to explore more fully the effects of intervention duration for compassion-based SEL, given that research on adult populations seems to demonstrate that intervention dosage is important. Concerningly, as the school-based SEL meta-analyses reported above appear to show no effects of intervention dosage on programme or intervention effectiveness, this could lead to reduced time investment in SEL curricula. This is especially true in countries such as the UK, the US, and China, where academic performance is valued above SEL. For example, in the UK, English, Maths, Science, Computing, Physical Education, and Citizenship (e.g., financial literacy, human rights, and political participation) are regulated National Curriculum statutory subjects, but Personal, Social, Health, and Economic (PSHE) education is not. Indeed, as PSHE, which covers SEL, is non-assessed and largely non-statutory in the UK, what is taught and the quantity (as well as quality) of such can vary widely from school to school. The same is true in the US. However, to what extent intervention dosage affects compassion-based SEL effectiveness has not yet been established. This is important to investigate, as pressured academic timetables can lead to the removal of, or reduction in time spent on, non-statutory subjects.

Therefore, the objectives of this research were two-fold. The first objective was to explore the effectiveness of the CMT lessons with younger children, specifically those aged 10–11. The second objective was to investigate whether reducing programme length by compacting session/lesson delivery affects effectiveness. Hence, in the first study, we extended the trial of the CMT lessons––hereafter referred to as Wellbeing: Our Brains, Our Bodies (WoBoB)—to primary pupils aged 10–11. In the second study, we repeated the WoBoB lessons with a further cohort of secondary school pupils aged 11–12 but used a compacted ‘intervention’ timeframe.

## 2 Methods

### 2.1 Design

A mixed methods convergent parallel approach, with both quantitative and qualitative data collected, was used for this research. Sample size was calculated based upon the quantitative element and the randomised cluster-control design used for each study. Using G*power (Faul et al., 2007), to obtain an interaction effect for each mixed-factorial design, with time (pre; post) and group (experimental group receiving WoBoB; control group receiving standard PSHE) as the independent variables, a medium effect size (Cohen’s *f* = 0.25) and acceptable power (i.e., 0.9; with alpha set at 0.05, two-tailed), the calculated sample size required was 46. Thus, recruitment of approximately 50–60 pupils per study was considered a sensible medium when considering standard class sizes, potential attrition, and ethical implications of over-recruiting (Hemming et al., 2018). Ethical approval for the study was granted by the University of Derby (College of Health, Psychology and Social Care), with three-level consent (School, Teachers, Pupils, respectively).

### 2.2 Study 1

#### 2.2.1 Participants

Sixty pupils in Year 6 (aged 10–11 years; 23 females; 35 males; 2 prefer not to say) from a two-form entry school participated in the study. Pupils in one form were assigned to WoBoB (30 pupils; 11 female, 18 male, 1 prefer not to say) and pupils in the second to standard PSHE (30 pupils; 12 females; 17 males; 1 prefer not to say).

For Study 1, assignment of classes to WoBoB (experimental) versus PSHE (control) was random.

#### 2.2.2 Procedure

##### 2.2.2.1 Wellbeing: our brains, our bodies (WoBoB)

WoBoB is a revised version of CMT-Pupils (Maratos et al., 2024), consisting of six lessons, each lasting approximately 40–50 min, conducted over one school half-term (6 weeks). It comprises expert-informed SEL materials designed for universal class delivery. In Study 1, lessons were led by FM, a doctoral psychologist specialising in emotion research. FM had over 18 years’ experience of leading emotion- and compassion-based initiatives and research; and led the development of WoBoB. Lesson delivery was supported by JH, a counsellor by profession, trained in the delivery of the approach. JH had over 5 years’ experience of delivering wellbeing interventions in a school setting.

A brief description of lesson content is provided in Supplementary Table 1 and the lesson delivery and evaluation schedule in Table 1 (Column 1).

**Table 1:** Lesson delivery and evaluation timeline.

Study 1 timeline	Study 2 timeline
Week 1: Baseline (T1) Measures	N/A
Week 2: Lesson 1	N/A
Week 3: Lesson 2	Week 1: Baseline (T1) Measures, Lesson 1
Week 4: Lesson 3	Week 2: Lesson 2, Lesson 3
Week 5: Lesson 4	Week 3: Lesson 4, Lesson 5
Week 6: Lesson 5	Week 4: Lesson 6, Post (T2) Measures
Week 7: Lesson 6	N/A
Week 8: Post (T2) Measures	N/A

##### 2.2.2.2 Standard PSHE

The standard (control) PSHE also consisted of six lessons, each lasting approximately 40–50 min, conducted over one school half-term (6 weeks). These materials were delivered by the Year 6 class teacher as they formed part of the school’s standard primary-aged PSHE provision as mandated by the UK government (Department for Education, 2021). They covered a range of topics, including 1. Understanding positive relationships, with particular reference to friendships, family relationships, and relationships with other children and adults. 2. Developing attributes such as self-belief and self-worth, honesty, integrity, courage, humility, kindness, generosity, trustworthiness, and a sense of justice. 3. Understanding the link between good physical health and good mental wellbeing. 4. Learning about different types of emotions. 5. Online safety. 6. Key facts about adolescence.

#### 2.2.3 Data collection and measures

To investigate engagement with, acceptability of, and reach of WoBoB, at the end of each lesson pupils completed a single-sided anonymous feedback form with six questions.

To investigate the wellbeing effects of WoBoB, a questionnaire pack, containing seven standardised measures, was completed by all pupils at two time points. Timepoint one (T1) was exactly 1 week before (i.e., the same day and time) pupils took part in either WoBoB or PSHE as usual. Timepoint two (T2) was exactly 1 week post Lesson 6 for both curricula. Cronbach’s alpha was taken as a measure of questionnaire reliability (i.e., internal consistency) at T1. Cronbach’s alpha allows for an understanding of how well multiple questions measure the underlying questionnaire concept. If these concepts are not reliably measured at T1, then results may be unreliable. Often, in programmes and interventions, Cronbach’s alpha can increase from T1 to T2. This is because concept introductions and discussions within the programme may improve familiarity with questionnaire wording. Thus, in child research, where certain concepts (and related scale wording) may be new, it is important to report Cronbach’s alpha at T1. At T2, pupils assigned to the WoBoB condition also answered up to four open-ended questions to explore the effects of the lessons more comprehensively.

##### 2.2.3.1 Child and adolescent perfectionism scale (CAPS)

The CAPS (Flett et al., 2016) consists of 22 items structured around two dimensions: Self-Oriented Perfectionism (SOP; 12 items), which measures motivation and efforts to be a perfectionist as well as the tendency to self-criticise; and Socially Prescribed Perfectionism (SPP; 10 items), which captures beliefs about perfectionist demands of the environment. A 5-point Likert scale (from 1 = *not true of me at all* to 5 = *very true of me*) is used for responses. Higher scores reflect greater perfectionism. Ashra et al. (2021) identified the CAPS as having strong psychometric properties. For the present study, *α* = 0.77 for SOP and *α* = 0.77 for SPP at T1 suggesting acceptable reliability for both subscales.

##### 2.2.3.2 Moods and feelings questionnaire—short version (MFQ-SF)

The MFF (Messer et al., 1995) consists of 13 items measuring how a child has been feeling or acting recently. Children are asked if a sentence is true about themselves using a 3-point Likert scale (from *not true* = 0, *sometimes* = 1, and *true* = 2). Higher scores reflect a tendency towards a depressed state. Internal reliability of the scale in New Zealand adolescents showed that Cronbach’s alphas were excellent for each time point (*α* = 0.91 to 0.93). For the present study, Cronbach’s *α* = 0.87 at T1 suggesting good reliability.

##### 2.2.3.3 Perceived stress scale–child (PSS-C)

The PSS-C (White, 2014) consists of 13 items that measure the perception of stress, including feelings of being rushed or worried, pressures associated with academic performance, friendships, and parents, etc. Children are asked to pick the answer that best describes their thoughts and feelings during the past week using a four-point Likert scale (where *never* = 0 and *a lot* = 3). Higher scores reflect a tendency towards greater perceived stress and anxiety. Internal consistency of this scale is reported as acceptable (*α* = 0.78, Marie et al., 2022). For the present study, Cronbach’s *α* = 0.69 at T1 suggesting questionable or marginal reliability.

##### 2.2.3.4 Rosenberg self-esteem scale (RSE)

The RSE (SRAlab.org, 2014) is a 10-item self-report measure of global self-esteem. It consists of items capturing overall feelings of self-worth and self-acceptance. For each statement, children are asked to report their general feelings towards themselves using a 4-point Likert scale (with responses coded as strongly agree, agree, disagree, or strongly disagree). Higher scores reflect greater self-esteem and feelings of self-worth. Both test/retest reliability (0.88, Robins et al., 2001) and internal consistency (*α* = 0.84–0.86, Tinakon and Nahathai, 2012) are reported as good. For the present study, mean Cronbach’s *α* = 0.79 at T1 suggesting acceptable reliability.

##### 2.2.3.5 Self-compassion scale for children (SCS-C)

The SCS-C (Sutton et al., 2018) consists of 12 items measuring positive and negative compassion. Children are asked to read the statements and click on the answer that applies most to how they usually feel using a 5-point Likert scale (from 1 = *Never* to 5 = *Always*). The questionnaire has a two-factor structure, with higher scores on the positive self-compassion subscale reflecting greater self-compassion and higher scores on the negative self-compassion subscale reflecting a tendency towards psychopathology and negative feelings towards the self. Sutton et al. (2018) reported that both subscales have acceptable internal consistency (Cronbach’s *α* = 0.81–0.83). For the present study, Cronbach’s *α* = 0.74 for the positive self-compassion subscale and *α* = 0.83 for the negative self-compassion subscale at T1, suggesting acceptable and good reliability, respectively.

##### 2.2.3.6 Trait anxiety inventory for children (STAIC-T)

The STAIC forms part of the wider State–Trait Anxiety Inventory for Children (Spielberger et al., 1973). The T-Anxiety scale measures relatively stable individual differences in anxiety proneness; that is, differences between children in their tendency to experience anxiety states. Children are asked to read sentences and decide, using a 3-point Likert scale (1 = *hardly ever*, 2 = *sometimes*, and 3 = *often*), which statement is true or best describes them. Higher scores reflect higher trait anxiety. The STAIC is considered the gold standard child anxiety measure, with established clinical validity (Kirisci et al., 1997). For the present study, Cronbach’s *α* = 0.89 at T1 suggesting good reliability.

##### 2.2.3.7 Warwick-Edinburgh mental wellbeing scale (WEMWBS)

The WEMWBS (Stewart-Brown et al., 2009) is a 14-item positively worded questionnaire designed to measure mental wellbeing and feelings. Children are asked to describe the feelings and thoughts that best represent their experiences over the past 2 weeks, using a 5-point Likert scale (where 1 = *none of the time* and 5 = *all of the time*). Higher scores indicate greater positive wellbeing. Clarke et al. (2011) reported that the WEMWBS has strong internal consistency (*α* = 0.87) and acceptable test–retest reliability (0.66). For the present study, Cronbach’s *α* = 0.82 suggesting good reliability.

##### 2.2.3.8 Open-ended questions (WoBoB condition only)

The four open-ended questions were designed to more fully access the lived/everyday experiences of pupils assigned to the WoBoB emotion and compassion lessons. The questions focused on how the WoBoB lessons may have affected: (i) how the pupil was feeling, their understanding of emotions, or their general wellbeing; (ii) their behaviour, including whether they had tried or were using the practices introduced; and (iii) their class, including how they behave with others and vice versa. The final question (iv) probed what they enjoyed most or would have liked more or less of. The actual questions used can be found in the Supplementary materials but were the same as those used by Maratos et al. (2024).

### 2.3 Study 2

#### 2.3.1 Participants

Fifty pupils in Year 7 (aged 11–12 years; 29 females; 21 males) from two separate forms in a seven-form entry school participated in the research. Pupils in one form were assigned to WoBoB (25 pupils; 15 females; 10 males) and pupils in a second form (25 pupils; 14 females; 11 males) to standard PSHE.

For Study 2, the assignment of classes to WoBoB (experimental) vs. PSHE (control) was random.

#### 2.3.2 Procedure

##### 2.3.2.1 Wellbeing: our brains, our bodies (WoBoB)

This was the same as for Study 1, with the exception that the six lessons were conducted over a period of four weeks, with each lesson lasting approximately 40–50 min (see Table 1, Column B). The lessons were again led by FM, but this time supported by CH. CH is a senior lecturer and chartered psychologist, trained in the delivery of the approach.

##### 2.3.2.2 Standard PSHE

The standard (control) PSHE also consisted of six lessons, each lasting approximately 40–50 min, conducted over the same 4-week period. These materials were delivered by the Year 7 class teacher as they formed part of the school’s standard secondary-aged PSHE provision as designated by the UK government (Department for Education, 2021) and are detailed in Maratos et al. (2024).

#### 2.3.3 Data collection and measures

The investigation of engagement with, acceptability, and reach of WoBoB was the same as for S1, with the exception that data were only collected at four time points: the end of lessons 1, 3, 5, and 6 (refer again to Table 1).

The process for investigating the wellbeing effects of WoBoB was the same as for Study 1, with the exception that T1 was immediately before the delivery of Lesson 1 and T2, including the open-ended questions, was immediately after the delivery of Lesson 6. Cronbach’s alpha scores were as follows:

##### 2.3.3.1 Child and adolescent perfectionism scale (CAPS)

*α* = 0.80 and SPP *α* = 0.74 at T1 suggesting good and acceptable reliability, respectively.

##### 2.3.3.2 Moods and feelings questionnaire (MFQ)

*α* = 0.85 at T1 suggesting good reliability.

##### 2.3.3.3 Perceived stress scale (PSS)

*α* = 0.72 at T1 suggesting acceptable reliability.

##### 2.3.3.4 Rosenburgh self-esteem scale (RSE)

*α* = 0.77 at T1 suggesting acceptable reliability.

##### 2.3.3.5 Self-compassion scale for children (SCC)

Positive subscale *α* = 0.73 and Negative subscale *α* = 0.85 at T1 suggesting acceptable and good reliability, respectively.

##### 2.3.3.6 Trait anxiety inventory for children (STAIC-T)

***α* =** 0.90 at T1 suggesting excellent reliability.

##### 2.3.3.7 Warwick-Edinburgh mental wellbeing scale (WEMWBS)

*α* = 0.87 at T1 suggesting good reliability.

##### 2.3.3.8 Open-ended questions (WoBoB condition only)

These were the same as in Study 1.

## 3 Data analyses

### 3.1 Study 1

#### 3.1.1 Intervention quality data

For the Likert scale data, frequency tables were produced, and descriptive statistics were used to explore the data. Ratings of 1–3 were coded as poor, 4–7 as average, and 8–10 as excellent.

#### 3.1.2 Quantitative measure data

For the questionnaire data, an independent researcher, FP, who was not involved in lesson delivery nor a compassion or wellbeing expert, and who was further unaware of all research objectives, analysed the data. Data were initially screened for biased responding and normality, including Q-plots, skewness, and kurtosis, revealing that deviations from normality were tolerable. Additionally, all Z scores fell between −3 and +3. Data were then matched by participant code. This led to a final sample size of 47: 27 in the experimental group receiving WoBoB and 20 in the control group receiving standard PSHE.

A mixed-factorial ANOVA, with time (T1, T2) as the within-subjects factor and group (experimental group receiving WoBoB; control group receiving standard PSHE) as the between-subjects factor, was used to explore each quantitative wellbeing measure. ANOVA is a well-accepted analysis procedure for controlled trial designs as it allows for the isolation of variability to determine the effects of a treatment. For any significant interaction, effects were explored via Bonferroni-corrected post-hoc pairwise comparisons, with partial Eta squared as the measure of effect size (i.e., magnitude of intervention effect for that specific measure).

#### 3.1.3 Qualitative data

All written responses were coded inductively for meanings and patterns using the six phases of thematic analysis developed by Braun and Clarke (2013, 2021). CH, an experienced mixed-methods researcher with extensive experience in progressing qualitative research with children (e.g., Harvey et al., 2020), led the analyses. Importantly, CH was not involved in the Year 6 lesson delivery or data collection and was further unaware of the quantitative results.

### 3.2 Study 2

#### 3.2.1 Intervention quality data

For the Likert scale data, frequency tables were produced, and descriptive statistics were used to explore the data. Ratings of 1–3 were coded as poor, 4–7 as average, and 8–10 as excellent.

#### 3.2.2 Quantitative measure data

For the questionnaire data, the independent researcher, FP, again analysed the data. Data were initially screened for biased responding and normality, including Q-plots, skewness, and kurtosis, revealing that deviations from normality were tolerable. Additionally, all Z scores fell between −3 and +3. Data were then matched by participant code, resulting in a final sample size of 39: 20 in the WoBoB group and 19 in the PSHE group.

As with Study 1, mixed-factorial ANOVA, with time (T1, T2) as the within-subjects factor and group (experimental group receiving WoBoB; control group receiving standard PSHE) as the between-subjects factor, was used to explore each quantitative wellbeing measure.

#### 3.2.3 Qualitative data

All written responses were coded inductively for meanings and patterns using the six phases of thematic analysis developed by Braun and Clarke (2013, 2021). JH, who has considerable experience in progressing qualitative research with children (e.g., Hurst, 2026), led the analyses. Importantly, JH was not involved in the Year 7 lesson delivery or data collection and was, furthermore, unaware of the quantitative results.

## 4 Results

### 4.1 Study 1

#### 4.1.1 Pupil evaluation of initiative (including acceptability and reach)

In total, 163 evaluation forms were completed across the six WoBoB lessons. This comprised between 25 and 30 forms completed per lesson. Descriptive data pertaining to pupil evaluation of each specific WoBoB wellbeing lesson are presented in Table 2. This demonstrated that the majority of pupils found each lesson excellent (mean 88%), as well as rated every lesson highly in terms of both enjoyableness (mean 77%) and helpfulness (mean 80%). Additionally, on average, 67% of pupils thought that the lessons would be useful for all Year 6 pupils; while 30% were unsure, only 3% of pupils indicated that the content would not be useful for all. This was in respect to Lesson 3 (1 child), Lesson 4 (2 children), Lesson 5 (1 child), and Lesson 6 (1 child).

**Table 2:** Primary pupil ratings of WoBoB on a lesson-by-lesson basis and overall frequency counts (and percentages).

Rating	Lesson 1	Lesson 2	Lesson 3	Lesson 4	Lesson 5	Lesson 6	Overall (%)
*How did you find the lesson overall?*							
Poor (1–3)	0	1	0	0	0	0	1 (0)
Average (4–7)	3	3	7	0	3	2	18 (11)
Excellent (8–10)	22	23	21	26	24	28	144 (88)
*How enjoyable did you find today’s lesson?*							
Poor (1–3)	0	0	0	0	0	0	0 (0)
Average (4–7)	9	4	7	8	6	3	37 (23)
Excellent (8–10)	16	23	21	18	21	27	126 (77)
*How helpful did you find today’s lesson?*							
Poor (1–3)	1	1	2	0	3	0	7 (4)
Average (4–7)	8	2	4	7	3	3	26 (16)
Excellent (8–10)	15	24	22	19	21	27	128 (80)
*Do you think today’s lesson would be useful for all Year 6 pupils?*							
Yes	20	19	17	15	15	28	114 (67)
No	0	0	1	2	1	1	5 (3)
Do not Know	5	8	10	9	11	7	50 (30)

#### 4.1.2 Quantitative wellbeing measures

For all measures, descriptive statistics are presented in Table 3. For the STAIC-T, the mixed-factorial ANOVA revealed a main effect of time (*F*(1,39) = 18.46, *p* < 0.001, ηp2 = 0.32), but more importantly, a Condition by Time Interaction (*F*(1,39) = 5.26, *p* = 0.027, ηp2 = 0.11). This interaction is displayed in Figure 1A and appears to demonstrate no difference in STAIT-T scores at T1, but a decrease in STAIC-T scores for the experimental group receiving WoBoB at T2 relative to T1. This was confirmed via Bonferroni-corrected post-hoc pairwise comparisons, showing a significant score decrease from T1 (*M* = 37.46, SE = 1.39) to T2 (*M* = 31.53, SE = 1.40) for the WoBoB group (mean difference = 5.92, p < 0.001, 95% CI = 3.72–8.1, ηp2 = 0.43), while for the control group, no significant difference in STAIC-T score from T1 to T2 was observed (*p* = 0.21). Levene’s test indicated that the assumption of homogeneity of variances was met at T1, *F*(1,39) = 1.14, *p* = 0.29, and at T2, *F*(1,39) = 0.83, *p* = 0.28.

**Table 3:** Mean (and standard deviation) scores for the questionnaire measures as a function of time and RC_L_T condition for 10–11-year-old primary school children.

Measure	Condition	Assessment time	
		Time 1	Time 2
CAPS-SOP	WoBoB PSHE	32.07 (6.90)	30.81 (9.69)
	Control PSHE	30.93 (8.77)	30.40 (7.58)
CAPS-SPP	WoBoB PSHE	20.63 (5.73)	19.74 (8.26)
	Control PSHE	21.67 (10.46)	22.87 (9.74)
MFQ-SF	WoBoB PSHE	20.41 (5.12)	17.85 (4.80)
	Control PSHE	20.00 (5.97)	19.43 (5.69)
PSS-C	WoBoB PSHE	25.92 (4.42)	23.74 (5.84)
	Control PSHE	23.67 (6.45)	23.87 (6.64)
RSE	WoBoB PSHE	28.15 (5.02)	29.63 (4.65)
	Control PSHE	28.00 (6.04)	28.33 (5.88)
SCS-C POS	WoBoB PSHE	**16.44 (4.66)** ^*+^	**18,41 (5.11)** ^**+**^
	Control PSHE	**20.60 (4.22)** ^*+^	**18.07 (5.81)** ^**+**^
SCS-C NEG	WoBoB PSHE	16.64 (4.77)	13.67 (6.59)
	Control PSHE	14.93 (6.91)	13.27 (5.88)
STAIC-T	WoBoB PSHE	**37.46 (6.63)** ^**+**^	**32.37 (8.03)** ^**+**^
	Control PSHE	**36.87 (7.84)**	**35.07 (7.61)**
WEMWBS	WoBoB PSHE	**43.04 (7.73)** ^*+^	**47.38 (9.33)** ^**+**^
	Control PSHE	**49.64 (8.00)** ^*^	**44.60 (10.43)**

Bold values indicates measures where time (1, 2) by group (Control Compassion) significant interactions were observed (*p* < 0.05). *Indicates significant between-group differences, ^+^Indicates within-group differences, for these significant interactions.

**Figure 1:**
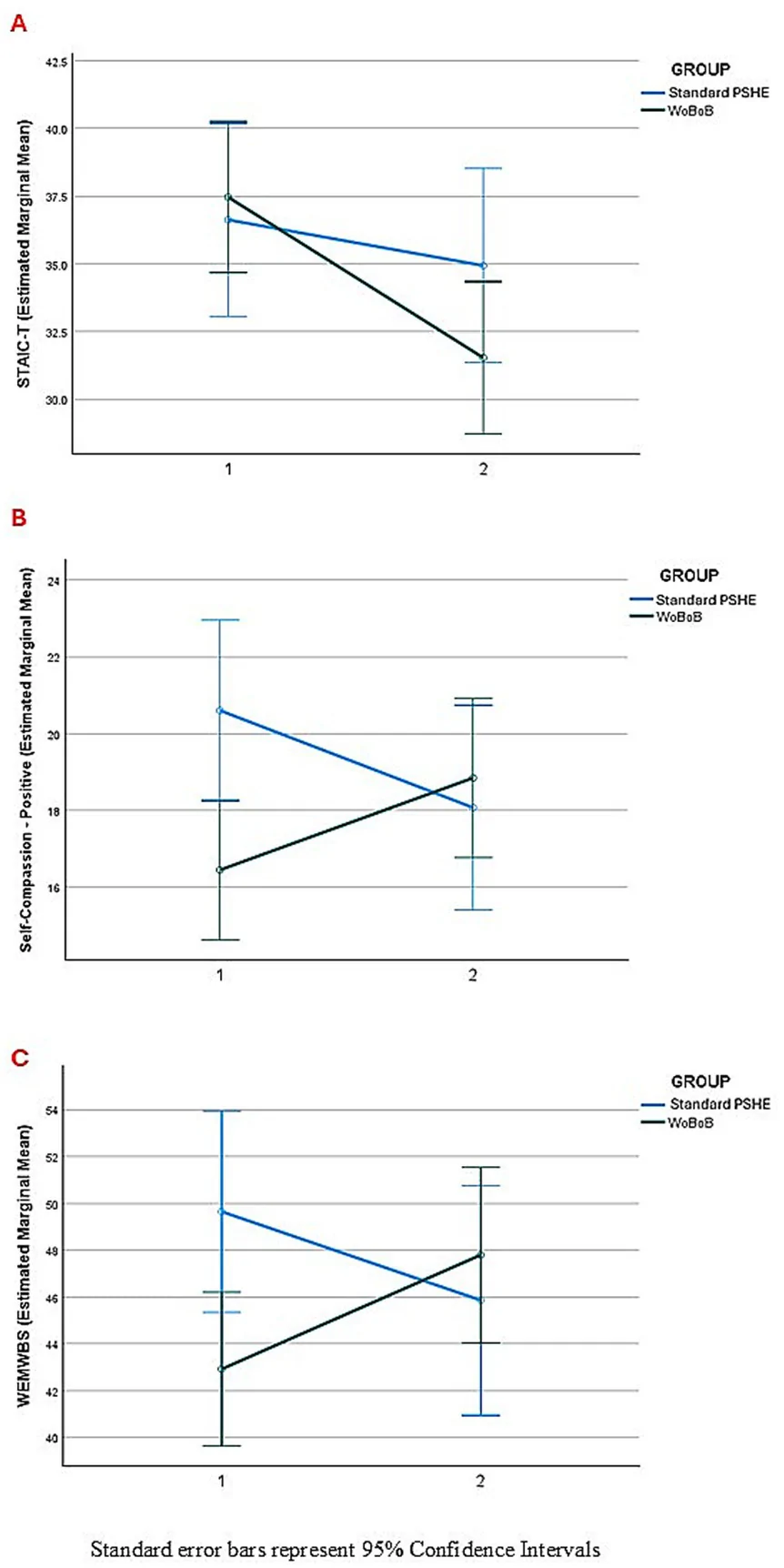
Group by time interactions for **(A)** Trait anxiety; **(B)** Positive self-compassion and **(C)** General wellbeing.

For the positive self-compassion subscale measure, analyses revealed a significant Condition by Time Interaction (*F*(1,38) = 9.74, *p* = 0.003, ηp2 = 0.20). This interaction effect is depicted in Figure 1B and demonstrates that whereas positive self-compassion increased for the experimental group receiving WoBoB from T1 to T2, for the control group receiving standard PSHE, the opposite effect was observed, that is, positive self-compassion decreased from T1 to T2. Bonferroni-corrected post-hoc pairwise comparisons showed that the control PSHE group (*M* = 20.60, SE = 1.16) and the experimental WoBoB group (*M* = 16.40, SE = 0.90) differed significantly in their positive self-compassion scores at T1 (mean difference = 4.16, *p* = 0.007, 95% CI = 1.18–7.14, ηp2 = 0.17), but by T2, the experimental group receiving WoBoB had experienced a significant increase (*M* = 18.84, SE = 1.02, mean difference = 2.40, *p* = 0.18, 95% CI = 0.44–4.35, ηp2 = 0.13), while the control group receiving standard PSHE had experienced a borderline significant decrease (*p* = 0.050). The two groups’ scores at T2 did not differ significantly (*p* = 0.64). Levene’s test indicated that the assumption of homogeneity of variances was met at T1, *F*(1,38) = 0.07, *p* = 0.78, and at T2, *F*(1,38) = 0.96, *p* = 0.80.

For the WEMWBS, analyses revealed a significant Condition by Time Interaction (*F*(1,36) = 10.51, *p* = 0.003, ηp2 = 0.22). This interaction effect is depicted in Figure 1C and demonstrates that whereas wellbeing increased for the experimental group receiving WoBoB from T1 to T2 (*M* = 42.91, SE = 1.61 vs. *M* = 47.79, SE = 1.85, mean difference = 4.87, *p* = 0.005, 95% CI = 1.58–8.16; ηp2 = 0.20), for the control group receiving standard PSHE, the opposite effect was observed, that is, wellbeing decreased from T1 to T2, albeit only marginally (*p* = 0.083). The two groups’ scores differed significantly at T1 (mean difference = 6.72, SE = 2.66, *p* = 0.016, 95% CI = 1.32–12.13; ηp2 = 0.15), but not at T2 (*p* = 0.530). Levene’s test indicated that the assumption of homogeneity of variances was met at T1, *F*(1,36) = 0.05, *p* = 0.82, and at T2, *F*(1,36) = 0.14, *p* = 0.70.

For all further measures, analyses returned no significant or marginally significant interaction effects. However, for the MFQ, a main effect of time was observed (*F*(1,41) = 7.87, *p* = 0.008, ηp2 = 0.16). This main effect reflects that for the MFQ, scores for all pupils decreased from Time 1 to Time 2. In this case too, Levene’s test indicated that the assumption of homogeneity of variances was met at T1, *F*(1,41) = 0.27, *p* = 0.60, and at T2, *F*(1, 41) = 0.86, *p* = 0.35.

#### 4.1.3 Qualitative analyses

Out of 27 children present at T2 data collection, 26 responded to open-ended question (OEQ) 1, 25 responded to OEQ 2, 26 responded to OEQ 3, and 26 responded to OEQ 4, rendering sufficient data for thematic analysis by CH. CH assimilated all written responses into a single document and removed any potentially identifying data to preserve participant and trainer confidentiality. CH then conducted thematic analysis (TA) based on the guidance of Braun and Clarke (2013, 2021). TA provides a systematic approach when aiming to identify patterns across a dataset. CH first read and re-read the document several times. Rather than coding the data for the four individual questions, CH generated initial codes in response to the overarching question *“What was the impact of WoBoB on child and peer wellbeing and behaviours?.”* Data were coded with an emphasis on data-driven codes, which are semantic in nature as opposed to latent (Braun and Clarke, 2013). From this, 11 codes were generated (see Supplementary materials). CH then grouped these 11 codes together to potentially represent four overarching conceptual themes. Following further review of the initial codes and patterns present in the data, CH finalised theme codes, titles, and quotes, and sent these to the lead author for final reflection. Changes to the TA at this stage by the lead author were minimal and simply involved very minor amendments to the theme titles.

The overarching message of the qualitative data related to the positive impact of the WoBoB lessons for the pupils. Their responses demonstrated that the pupils developed a deeper understanding of their own emotions, and those of others, and that they now had tools and techniques which they could put into practice to help regulate their emotions when needed. The beneficial impact of this approach is demonstrated across all four themes. Below, an overview of the themes is presented.

##### 4.1.3.1 Theme 1: increased understanding of emotions—of self and others

In this theme, the pupils demonstrated an awareness of how the lessons had helped them to understand their own emotions and those of others, with some pupils developing the skill of introspection.

Children were well able to demonstrate their growing emotional literacy.

*“In the well-being lessons it has made me discover lots of emotions and different ones I’ve never felt before.”*

*“…it reminds me of what’s inside and how I feel.”*

Several pupils also noted how their understanding of their classmates’ emotions had improved because of the lessons:

*“The well-being lessons have been very helpful of understanding other people’s emotions and how they are feeling.”*

*“It helped me understand how others feel sometimes and how they act.”*

Some pupils were also able to apply their learning one step further, demonstrating insight into how other people may also experience similar emotions to themselves:

*It has helped me understand that everyone has bad days and that it’s not just me.*

Thus, this theme demonstrates how WoBoB increased children’s emotional literacy and understanding of not only their own emotions and those of others, but in some cases, the higher-level cognitive skill of perspective-taking and the idea of a shared common humanity.

##### 4.1.3.2 Theme 2: appreciation of practices to regulate emotions

The majority of pupils were able to articulate that they had used the techniques and practices introduced across the WoBoB lessons.

*“I have used calming imagery.”*

*“Calm place and hand breathing.”*

*“I’ve been doing the regal walking.”*

Many of the children were further able to share examples of how they used the different techniques taught in the lessons, some as a general strategy for wellbeing and calmness in respect to their own emotions:

“*The calm place imagery has helped me with my sleep and falling asleep faster.”*

*“The compassionate imagery has helped me calm down a lot.”*

*“I use the hand breathing to help me calm down.”*

*“I think this has helped a lot with when I ether got angry I try to do my best at calming down and then go of and try to do the hand breathing.”*

Others were able to recognise that they now had a range of resources they could use to help them cope with difficult emotions, especially in the context of inter-relationships and social situations.

*“The imagery has helped to calm me down when my brother has stressed me out.”*

*“I did calm place imagery when there was a lot of people and it was loud.”*

*“Clam place imagery has helped me the most in situations and hand breathing i have a brother why can get mad a little more easy more than other people so if we argue I go in my room and do one of those sessions.”*

Thus, this theme highlighted that pupils were able to extrapolate the techniques taught in lessons to their wider behaviour and lives. They were able to understand when the techniques were useful to apply, and they were able to verbalise the impact these techniques had on their emotions, ultimately demonstrating their growing emotional regulation skills.

##### 4.1.3.3 Theme 3: Prosociality through the principles of compassionate mind training and compassionate flow

This theme evidences that the children were able to understand foundational principles of compassionate mind training, and that compassion involves different flows: compassion to self, compassion to others, and compassion from others.

*“I liked how we went around the class and wrote nice things about each other to the class. it made me feel positive about myself and hopefully made others feel the same too.”*

*“Yes it has made me be kinder to myself and relax more also showing compassion to others.”*

“*I think it is important to love your self and others for example class mates friend family*.”

Some pupils were further able to articulate how this related to psychoeducation theory:

*“Listening to everyone’s weekend (drive, soothing, threat intro).”*

For others, it was clear that the principles of compassionate flow had been embedded, even if they struggled to articulate them.

*“Yes it has helped me understand more of what people mean to me and what I mean to them it has helped my emotions.”*

Therefore, despite the younger age of the pupils (i.e., 10–11 years), this theme clearly demonstrates that children can grasp the concepts of compassion and compassionate mind training and their benefits for self and others.

##### 4.1.3.4 Theme 4: pleasure in learning

The final theme reflects how the children expressed their enjoyment of the lessons. There were many examples of specific activities that had a positive impact, with pupils expressing their pleasure in those activities:

*“I like that we did the regal walking.”*

And often, the impact of those activities on their experiences. For example,

*“I loved the calming imagery it made me feel relaxed.”*

*“I like all the breathing practices and practices to keep calm it was very useful for me.”*

Other pupils expressed their overall enjoyment derived from engaging in the lessons:

*“I enjoyed every moment of it. It made me feel good.”*

*“I loved the well-being lessons it helped me very much.”*

*“I enjoyed it all because it was amazing.”*

However, it should be noted that a minority of pupils expressed that the lessons had not had an impact on their behaviour:

“*not really*.”

“*Not really but it has made me more cheeful*,”

Or that of the wider class, for example,

“*not really*.”

“*Not really I’ve noticed some people are more talkative though.”*

In sum, this theme reflects the enjoyment derived by most of the pupils when taking part in the lessons. They were able to identify specific practices or content that had particularly stood out for them as pleasurable, while others reflected on how all of the lessons had been rewarding and a positive experience for them.

### 4.2 Study 2

#### 4.2.1 Pupil evaluation of initiative (including acceptability and reach)

In total, 93 evaluation forms were completed across the six WoBoB lessons. This comprised between 22 and 25 forms completed per lesson. Descriptive data pertaining to pupil evaluation of each specific WoBoB lesson are presented in Table 4. This demonstrated that the majority of pupils found each and every lesson excellent (mean 56%), as well as excellent in terms of both enjoyment (mean 55%) and helpfulness (mean 52%). Additionally, on average, 68% of pupils thought that the lessons would be useful for all Year 7 pupils, and while 31% were unsure, only 1% of pupils indicated that the content would not be useful for all. This was in respect to Lesson 1 (1 child).

**Table 4:** Secondary pupil ratings of WoBoB on a lesson-by-lesson basis and overall frequency counts (and percentages).

Rating	Lesson 1	Lesson 2/3	Lesson 4/5	Lesson 6	Overall (%)
*How did you find the lesson overall?*					
Poor (1–3)	1	0	0	1	2 (2)
Average (4–7)	8	13	11	7	39 (42)
Excellent (8–10)	14	10	14	14	52 (56)
*How enjoyable did you find today’s lesson?*					
Poor (1–3)	1	1	0	0	2 (2)
Average (4–7)	9	12	8	10	39 (42)
Excellent (8–10)	13	9	17	12	51 (55)
*How helpful did you find today’s lesson??*					
Poor (1–3)	2	1	2	1	6 (7)
Average (4–7)	9	13	9	7	38 (41)
Excellent (8–10)	11	9	14	14	48 (52)
*Do you think today’s lesson would be useful for all Year 7 pupils?*					
Yes	13	14	19	17	63(68)
No	1	0	0	0	1 (1)
Do not Know	9	9	6	5	29 (31)

#### 4.2.2 Quantitative wellbeing measures

For all measures, descriptive statistics are presented in Table 5. Mixed-factorial ANOVAs, with condition (experimental group receiving WoBoB; control group receiving standard PSHE) as the between-subject variable and Time (T1, T2) as the repeated-subjects variable, were conducted for each dependent questionnaire variable.

**Table 5:** Mean (and standard deviation) scores for the Wellbeing Questionnaire measures as a function of time and RC_L_T condition for 11–12-year-old secondary school pupils.

Measure	Condition	Assessment time	
		Time 1	Time 2
CAPS-SOP	WoBoB PSHE	33.15 (8.20)	34.50 (9.32)
	Control PSHE	35.21 (8.93)	35.88 (8.65)
CAPS-SPP	WoBoB PSHE	23.65 (6.90)	25.25 (7.53)
	Control PSHE	21.84 (5.71)	24.24 (7.28)
MFQ-SF	WoBoB PSHE	21.45 (5.28)	22.30 (5.78)
	Control PSHE	20.21 (4.47)	20.29 (4.59)
PSS-C	WoBoB PSHE	27.30 (6.25)	26.75 (6.95)
	Control PSHE	25.42 (4.99)	24.65 (5.99)
RSE	WoBoB PSHE	27.20 (5.07)	26.15 (4.44)
	Control PSHE	28.37 (3.09)	27.41 (4.12)
SCS-C POS	WoBoB PSHE	18.40 (5.33)	17.70 (5.81)
	Control PSHE	20.37 (3.83)	19.06 (4.52)
SCS-C NEG	WoBoB PSHE	18.65 (6.02)	18.35 (5.67)
	Control PSHE	16.79 (5.24)	17.65 (5.09)
STAIC-T	WoBoB PSHE	39.20 (8.82)	40.45 (8.83)
	Control PSHE	38.95 (9.50)	39.06 (10.05)
WEMWBS	WoBoB PSHE	47.65 (10.94)	44.95 (9.24)
	Control PSHE	45.47 (6.94)	44.76 (10.15)

For all measures, analyses returned no significant or marginally significant interaction effects. The only result that trended towards significance was a main effect of time for the socially prescribed perfectionism sub-scale (*F*(1,37) = 2.99, *p* = 0.09, ηp^2^ = 0.07). This indicated that for all participants, socially prescribed perfectionism increased from Time 1 to Time 2.

#### 4.2.3 Qualitative analyses

Out of 22 children present at T2 data collection, 22 responded to OEQ 1, 22 responded to OEQ 2, 20 responded to OEQ 3, and 21 responded to OEQ 4, providing sufficient data for thematic analysis by JH. JH assimilated all written responses into a single document and removed any potentially identifying data to preserve participant and trainer confidentiality. JH then conducted thematic analysis (TA) based on the guidance of Braun and Clarke (2013, 2021). TA provides a systematic approach for identifying patterns across a dataset. JH first familiarised herself with the dataset through repeated reading of the pupils’ responses. Then, rather than coding the data for the four individual questions, JH generated initial codes in response to the overarching question “*What was the impact of WoBoB on child and peer wellbeing and behaviours*?” Through initial coding, JH generated 11 conceptual categories (see Supplementary materials), which were then iteratively reviewed across the full dataset. Conceptually related categories were combined and refined into four overarching themes. The final thematic structure and supporting quotations were reviewed with the lead author, with only minor refinements following discussion.

The overarching message of the qualitative data was that the lessons improved pupils’ emotional literacy, including understanding and acceptance of their own emotions and the emotions of others. Pupils were then able to utilise this knowledge for their own benefit, by judging when emotion regulation was needed and applying the techniques and practices taught in the lessons to move from one emotional state to another. They were also able to articulate the benefits of lesson content and associated practices across their class, leading to an improved classroom climate. Many pupils were also able to recognise the usefulness of the lessons in their everyday lives and how learning could be generalised. However, some pupils reported that engaging with the lessons was challenging, reflecting difficulties in concentrating or discomfort in discussing their own emotions in class. Below is a concise overview of the themes.

##### 4.2.3.1 Theme 1: increased understanding of emotions and perceived personal outcomes

This theme covered increased understanding of emotions, as well as acceptance and normalisation of emotions. Considering first emotional literacy, pupils frequently reported improved awareness of their own emotions and those of others. This was conveyed by the children stating the lessons.

*“…helped me understand mine and other people’s emotions.”*

*“I have learnt how to recognize if my friends are stressed.”*

*“…understand the way other people are feeling based of their facial expression.”*

They were also able to articulate how the content of the social emotional learning had directly impacted their own emotions:

*“theyve made me feel more relaxed and less stressed.”*

*“It has helped me to see the good part of life.”*

*“I think it has helped me relax more.”*

*“I think it had an impact on me as I have tried to look on the positive side of things and not stress as much.”*

Pupils could further describe a range of personal outcomes associated with the lessons. For example, they were able to accept that their emotional responses could be the same as, or differ from, others.

*“its okay to have differnt emotions and opinions to other people.”*

*“i have learnt everyone feels the same at one point in their life and its not just me.”*

*“yes I think it has helped. I think that I have learn everyone has felt the same way and to belive in my self.”*

*“Its okay to feel sad sometimes because youre never alone and someone is most likely to understand.”*

This suggests the pupils had gained an understanding of the idea of common humanity and perspective-taking.

Building upon their growing emotional literacy, several pupils also described being able to use the practices introduced, such as breathing, imagery, and regal walking, to manage stress, anger, or worry. Indeed, many pupils were able to link these strategies to behavioural change, including thinking more positively or controlling strong emotions.

*“its helped to brethe amd calm down.”*

*“the compassionate imaginary, calm place imaginary and the breathing tequnique has helped me.”*

*“The have made my behaviour better because I can now control what I do to keep my anger on the inside and to not let it out and hurt anybody.”*

*“I have used regal walking and deep breathing when I am stressed or trying to relaxed.”*

*“the breathing taking my mind of my worries.*

Taken together, pupils’ responses suggest that the wellbeing lessons were associated with a range of perceived personal outcomes, including increased understanding of emotions in the self and others, which then led to acceptance of emotions. This theme also demonstrates how pupils’ awareness of their emotions led to better emotion regulation through applying the techniques taught in the lessons to manage certain emotions.

##### 4.2.3.2 Theme 2: changes in the classroom environment

For the Year 7 pupils, a major impact of WoBoB was the classroom climate. Pupils described changes within their class following the wellbeing lessons, including increased calm, improved behaviour, more positive interactions, and greater openness. While some pupils identified specific behavioural changes related to the practices,

*“the breathing has carmed everyone down.”*

Others mapped these changes onto content:

*“i think that before pd some kids were being mean to each other but now some kids get on really well together and dont have fall outs and be mean to each other because they have learnt alot about being nice to other kids.”*

*“a lot of people have spoke how they feel and whats going on in their lifes and i didnt know.”*

While others still simply referred to a broader shift in the overall tone of the class:

*“My friends have become more friendly and people in my class are more friendly as well.”*

*“I think that my class has become much more calmer and not as loud when learning or speaking to their partners.”*

*“I think it kind of helped as I can see people in my class relax more and not worry as much.”*

*“I think it made the class a bit more open.”*

However, not all pupils reported noticing differences, indicating variability in how class-level changes were perceived. For example, one student reported:

*“i think it helped some people but not everyone”* and another that *“…everyone is mostly the same people as before.”*

In sum, the accounts of many pupils indicated that the lessons were associated with an improved classroom dynamic and a general improvement in the classroom climate. This included greater calmness, openness, and more beneficial peer interactions. However, a minority reported limited or no impact on their fellow classmates and therefore the classroom environment.

##### 4.2.3.3 Theme 3: pupil engagement—pleasure, enjoyment and areas for development

This theme overviewed what the pupils valued as well as what might have added to their positive experiences.

Pupils described their engagement with the WoBoB lessons, identifying particular activities and approaches that they valued. Some especially valued the practical and experiential elements such as breathing, imagery, relaxation, and movement-based exercises:

*“the brething activities.”*

*“I preferred doing more of the ‘relaxing’ when we would draw things that keep us calm or that help us relax.* e.g. *music, pets, friends.”*

*“I enjoyed the regal walking and the imaginary place because they calmed me down.”*

*“I liked doing the breathing because it helped me to feel relaxed and calm.”*

Whereas other students valued how the content could be extrapolated to their lives generally:

*“To be honest I really did enjoy these sessions and they helped me understand more about myself and how I can take care of my mental health more.”*

*“i really enjoyed the whole lessons the made me feel safe and calm during a really stressful time in my life.”*

One pupil even articulated how the learning environment supported their participation, making them feel safe and not pressured to contribute:

*“i liked how we werent pressured to have a say or particapate if the subject was uncomfortable.”*

Some students did suggest areas for development, which included wanting more active components:

*“yes i liked the walking methods i would like more of that.”*

*“probaly going outside for fresh air.”*

And more focus on emotions:

*“it would have been better if there was more talking about the drive, threat and soothing emotions.”*

Taken together, the majority of pupils’ responses reflected positive engagement with the wellbeing lessons, with many identifying specific elements they found helpful and supportive. At the same time, some pupils suggested ways the lessons could be extended, indicating a desire for more active content.

##### 4.2.3.4 Theme 4: personal and inter-personal challenges

While many pupils described positive outcomes associated with the wellbeing lessons, a minority reported difficulty engaging with certain aspects. Some of these challenges focused on difficulties with concentration and discomfort discussing personal feelings:

*“I cant focus on anything so the calm down dosent really work for me.”*

*“I do not like PSHE lessons that much because I do not like talking about myself a lot.”*

*“i have not really been able to concentrate when im doing those things and i never do them at home.”*

Other pupils simply reported few effects of the WoBoB lessons:

*“Some of it made me feel a bit relaxed, but other than that I did not feel any change on my behaviour.”*

Thus, this theme highlights that pupil experience of the WoBoB lessons was not uniform. Some pupils reported difficulties engaging with the approaches used or lesson content, and a minority further reported that the lessons were of limited benefit. These responses underscore the variability in how the lessons, and their value, were experienced across the pupils.

## 5 Discussion

A current focus of many governments is access to effective mental health and wellbeing support in schools (e.g., Department for Education, UK, 2021; Ministry of Health, New Zealand, 2021; Public Health Agency of Canada, 2021). However, concerns have been raised regarding the effectiveness of such interventions, including lack of theoretical grounding, poor-quality study design, and/or limited trials (Kuyken et al., 2023; Cary and Webb, 2025). In this study, we extended the trial of an emotion and compassion SEL lesson suite (‘WoBoB’) by exploring effectiveness: (1) in children of a younger age; and (2) with a shortened intervention ‘dosage’ (i.e., compacted delivery). Our reasoning for these extensions was, firstly, that there is now good evidence that for children aged 10–11, compassion-based SEL may protect against mental health deteriorations, promote good wellbeing, and encourage positive virtue formation. Secondly, we aimed to investigate the extent to which ‘intervention’ dosage affects compassion-based SEL effectiveness, as this has not yet been established. The first objective was addressed through Study 1, and the second objective through Study 2. Quantitative results revealed that while in Study 1 the effectiveness of the SEL lessons extended to younger children (aged 10–11), replication of effects in Study 2 with children aged 11–12 was negated when programme dosage (through compacted 4- vs. 6-week delivery) was reduced. Through both studies, qualitative results revealed benefits for all children in terms of emotional literacy, emotional regulation, and benefits of the lessons to the self and others. However, in Study 2, children demonstrated more variety in their responses. Study findings will be discussed in turn, before consideration of the benefits and limitations of high-quality small-scale trials and future directions.

Previously, Maratos et al. (2024) demonstrated that a compassion-based curriculum for pupils aged 11–12 was well-received and, compared to standard PSHE, protected against increases in anxiety. To meet objective 1, in Study 1, we progressed the emotion and compassion lessons (now referred to as Wellbeing: Our Brains, Our Bodies, or WoBoB) with younger children aged 10–11. Importantly, with this younger age group, the results replicated those of Maratos et al. (2024) and revealed that the lessons were well-received in terms of acceptance and reach, and also impacted anxiety. Additionally, we found that WoBoB, compared to standard PSHE, further impacted self-compassion and general wellbeing.

In considering first acceptability (including responsiveness) and reach, the results of Study 1 broadly replicated, if not exceeded, those of Maratos et al. (2024). To elaborate, most Year 6 pupils found WoBoB’s core curriculum content excellent (88% vs. 85% as reported by Maratos et al. (2024) and rated the lessons as excellent in terms of enjoyability [77% vs. 79% as reported by Maratos et al., 2024] and usefulness [80% vs. 67% as reported by Maratos et al., 2024]. Moreover, 67% of pupils [vs. 68% as reported by Maratos et al., 2024] suggested that the lessons would be useful to all pupils in Year 6 (i.e., those aged 10–11). The results of the qualitative analyses concurred with this quantitative data. For example, one theme that emerged from the qualitative data was ‘Pleasure in Learning’ (Theme 4). This theme captured children’s general expression of their enjoyment of the lessons and content. Thus, in respect to implementation effectiveness, the results of Study 1 demonstrate good extension of the acceptability and reach of the WoBoB curriculum to younger children, that is, those in upper primary/elementary school. This finding is important because high-quality implementation is argued to be an essential condition of any effective social and emotional learning programme and can affect the outcomes achieved (e.g., Wilde et al., 2019).

Like Maratos et al. (2024), we further found that, in Study 1, the WoBoB lessons impacted trait anxiety. However, while Maratos et al. (2024) found that in older children the lessons stabilised anxiety (i.e., prevented increases), we found that in younger children the lessons decreased child reports of trait anxiety from T1 to T2. To expand on this, results revealed that reports of trait anxiety significantly decreased from T1 to T2 for the WoBoB group (with a notably large effect size) but not for the standard PSHE group. Therefore, reductions in anxiety were only observed for the experimental group. We argue that this result reflects the content of WoBoB, which includes an understanding of emotion regulation as a core active ingredient. Furthermore, WoBoB is built upon a biopsychosocial approach to behaviour. This includes an understanding of threat, drive, and soothing emotions, acknowledging that these emotions are common to us all, as well as the introduction of practices to target emotion regulation through the activation of the parasympathetic nervous system (PNS) to regulate threat- and drive-related sympathetic nervous system (SNS) activity. This is important because SNS activity contributes to (hyper-)arousal, which underlies physiological anxiety (Aritzeta et al., 2022; Porges, 2017; Chalmers et al., 2014). Consistent with Maratos et al. (2024), we propose that a combination of relevant and scientifically accurate emotion psychoeducation allows children to accurately understand their emotional physiology. The introduction of practices to help children regulate SNS activity aids their understanding of emotion regulation and, consequently, the practice of worry/anxiety-dissipating thoughts, behaviours, and strategies when needed. The qualitative data corroborated this finding, revealing that children were able to express their growing emotional literacy with respect to understanding their own emotions (Theme 1) and, when needed, use the introduced practices to cope with difficult emotions or as a general strategy for wellbeing and calmness (Theme 2).

In Study 1, we found that WoBoB improved positive self-compassion and general wellbeing. Considering compassion first, it was found that while positive self-compassion decreased from T1 to T2 in children who had undertaken the standard PSHE, for those who had undertaken WoBoB, positive self-compassion significantly increased from T1 to T2. According to Levantini et al. (2025), the self-compassion scale for children measures a child’s ability to offer themselves kindness, recognise shared human experiences, and maintain awareness when facing failure, stress, or mistakes. Given that self-compassion psychoeducation and practices are a core component of WoBoB, including a focus on self-compassion practices, it is perhaps not surprising that the WoBoB group showed increases in positive self-compassion. However, this finding is important, because recent research has demonstrated that in adolescents aged 12–14, compassionate self-responding negatively predicts anxiety and depression scores, while uncompassionate self-responding positively predicts these (Nguyen et al., 2025). Therefore, working with children to introduce strategies to increase self-compassion through, for example, the introduction of WoBoB with primary-aged children may be very beneficial in protecting against future mental health problems. To this end, it is concerning that the control PSHE group demonstrated a borderline significant decrease (*p* = 0.05) in positive self-compassion from Time 1 to Time 2. Sadly, this aligns with the downward trends we are seeing in youth wellbeing more generally (e.g., Blanchflower et al., 2024; Dumuid et al., 2023), but demonstrates a pressing need to include more self-care, kindness, and compassion-based materials in SEL generally.

In considering the qualitative data, not only did the thematic analyses corroborate the emergence of (greater) self-compassion through the WoBoB curriculum, but also the ability of these younger children to perspective-take and apply compassion to others, as well as accept compassion from others. For example, Theme 3 of the thematic analyses demonstrated that children were able to understand and embed the principles of compassion for others and recognise the benefits of this, for example, the advantages of loving others. The flows of compassion (Gilbert, 2014) are a key tenet of WoBoB. As WoBoB increased each flow (and by extension prosociality), this has implications for moral concern and expansiveness. Given recent research (Marshall et al., 2025) has suggested that a child’s moral concern circle can begin to narrow from age 10, the current findings demonstrate that the practice of compassion can prevent this narrowing from occurring. Indeed, delivering WoBoB to children aged 10–11 may provide an ideal virtue-formation window, where the development of first-order theory of mind combined with malleable moral expansiveness allows for prosociality and positive virtue formation, to the benefit of all.

Finally, in Study 1, we found that wellbeing significantly increased from Time 1 to Time 2 for children who had undertaken WoBoB (with a large effect size noted), while for the standard PSHE it decreased, with this decrease trending towards significance. This result likely reflects the core emotion and compassion psychoeducation and practice content of WoBoB. This includes lessons dedicated to helping children understand their own and others’ emotions, as well as several practices and activities to help children better regulate their emotions and encourage all three flows of compassion. Additionally, and of key importance, an underlying premise of WoBoB is the application of a strengths-focused approach (see Seligman and Peterson, 2004). This directly opposes deficit-based models of wellbeing that are now often the foundation of school-based mental health interventions and programmes. In deficit-based models of wellbeing, the focus is on diagnosing symptoms and fixing individual flaws, concentrating on ‘what is wrong and needs to be fixed’ (e.g., Anglin and Polanco-Roman, 2017). The deficit-based approach has also been criticised for pathologising normal behaviours (Dinishak, 2022). For example, in deficit-based approaches, it is common to introduce children (and adults) to the concept of a ‘fight or flight’ system. However, in WoBoB, children are provided with accurate information about emotion physiology. We have an SNS that prepares us for action (e.g., Pinel and Barnes, 2021), whether that be fight, flight, or *fun* activities. This enables children to understand bodily responses such as ‘fast heartbeat’, ‘butterflies in one’s tummy’, and ‘sweaty hands’ as part of normal emotion physiology, rather than interpreting these physiological responses solely as indicators of anxiety, stress, anger, and worry. We argue that in WoBoB, this shift in approach to child wellbeing encourages children to move away from deficit-based thinking (e.g., “I have butterflies in my tummy I must be scared”) towards a more balanced understanding of bodily physiology and emotions (e.g., “I have butterflies in my tummy, I could be excited”), thereby promoting better wellbeing (see Figure 2) as well as reductions in anxiety.

**Figure 2:**
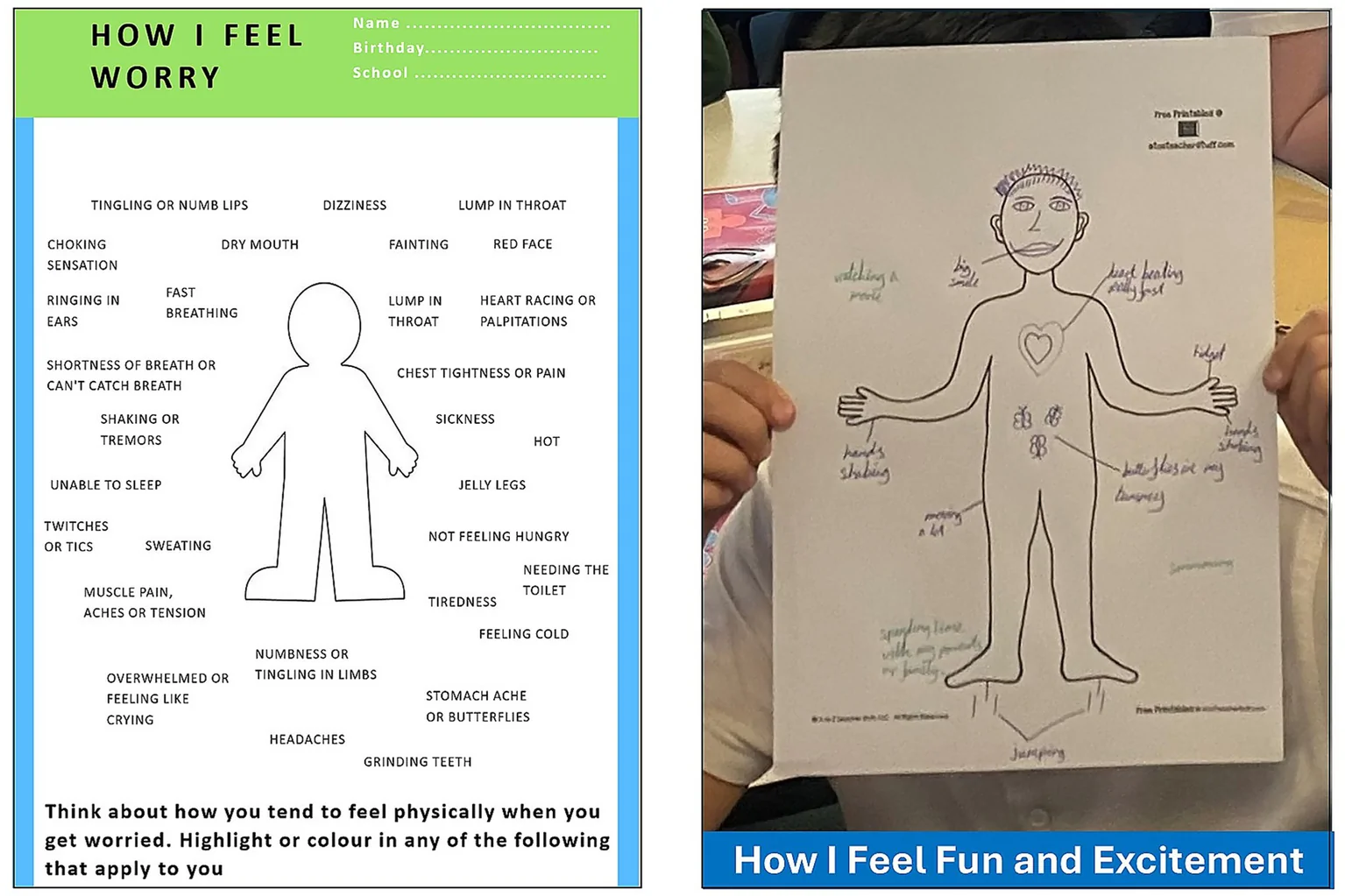
Re-education of the nation: Moving from the application of deficit- to strength-based approaches. Left panel: Open-access mental health provision for children aged 5 and over (reproduced from The Worry Toolkit, Cumberland Council, n.d., open access). Right panel: In WoBoB, children explore their own emotional physiology in response to both threat and drive (pictured) emotions. Through guided discovery, children learn that a variety of emotions are associated with sympathetic nervous system activity, not just those associated with ‘fight and flight’. Children are not primed to (mis)interpret and (mis)attribute all physiological responses to emotions of fear, worry, anger, etc.

The qualitative data appear to support this finding. For example, not only did many children report using regal walking, calming imagery, and soothing/hand breathing to help with their wellbeing when feeling angry or stressed (Theme 2), but many children also reported using these techniques for pleasure and enjoyment (Themes 2 and 4). Taken together, this strengths-based content and the children’s developing understanding of emotions and emotion regulation, as demonstrated via the thematic analyses, likely contributed to the quantitative increases in general wellbeing observed.

In Study 2, an exploration of programme dosage, it is important to note that we did not fully replicate the effects of Maratos et al. (2024) when the WoBoB lesson suite was conducted over a 4-week period. Considering first implementation data, despite 68% of children again suggesting the lessons would be useful to all pupils in Year 7, and whilst the majority of children still rated core curriculum content, enjoyability and usefulness as excellent (56%, 55% and 52%, respectively), these percentages were considerably lower than those Maratos et al. (2024) reported. Thus, these data suggest that implementation efficacy was impacted by compacted programme delivery. In accordance with this finding, and the arguments of Wilde et al. (2019) that high-quality implementation is an essential condition for achieving SEL outcomes, the Study 2 quantitative analyses further returned no significant results regarding the benefits of WoBoB over and above the standard PSHE. While it is possible that in Study 2, the results reflect the slightly smaller sample size and a problem of power, we would argue that what is essential for high-quality compassion-based SEL is time to embed learning, skills, and practices, along with the opportunity to practice these introduced skills and practices (see also Weare, 2015). This is simply not possible when the lessons are taught back-to-back. In these circumstances, opportunities for consolidating emotion regulation and compassionate responding learning, as well as repeated practice and ongoing reflection, are limited.

In support of these arguments, the qualitative thematic analyses revealed that while the lessons increased emotional literacy, including acceptance and normalisation of emotions (Theme 1), the children liked, and would have liked more of, the active practice elements (Theme 3). To expand, while Theme 1 highlighted increased understanding of emotions, quotes within this theme also highlighted how children were able to utilise the introduced practices to emotionally regulate. Adding to this, via Theme 3, it was evident that the children wanted more of this content (e.g., regal walking and emotional literacy). When the lessons are conducted over 6 weeks, this is possible given: (i) the weekly check-in activity, designed to target emotion and emotional regulation learning, and (ii) the weekly challenges (i.e., homework) set; both of these aspects are designed to increase emotion literacy skills and independent practice of the emotion regulation and compassion practices introduced. Condensing the curriculum over a shorter period removed opportunities for repetition of these activities and independent practice by over 50%. Consistent with this, Theme 4 revealed the children did not generalise the learning (e.g., ‘I never do them at home’) and/or struggled to concentrate on content and practices when the lessons were longer (i.e., 2 × 50 min consecutively). The Study 2 results, therefore, directly opposed the findings of Cohen et al. (2024). While Cohen et al. (2024) found that interventions consisting of ≤4 sessions still led to significant improvements in mental health/wellbeing compared to control conditions, Study 2 revealed that for compassion-based SEL, intervention dosage is important for implementation efficacy and changes in indices of wellbeing measured via quantitative questionnaires.

Indeed, as emotion regulation and compassionate self- and other-responding are skills that typically require repeated practice, ongoing reflection, and opportunities to revisit/consolidate before stable changes are seen (Gilbert and Procter, 2006; Maratos et al., 2019; Matos et al., 2022; Cefai et al., 2022), we argue that compassion-based SEL designed to impact wellbeing cannot be compacted by delivering sessions or lessons back-to-back. That said, it should be noted that aspects of the Study 2 qualitative data did reveal some benefits of WoBoB on class dynamics, the classroom environment, and peer interactions, as exemplified by Theme 3. This finding suggests that elements of prosociality and moral concern perhaps simply require thought and reflection, as opposed to repeated practice, and therefore can be introduced and taught over shorter dosages.

### 5.1 Limitations and considerations

One limitation of the present studies was the small sample sizes, which could, for Study 2, potentially have resulted in the null quantitative findings and a Type 2 error. However, we have provided counterarguments for this finding utilising the implementation, quantitative, and qualitative data. Thus, the more plausible explanation for the null Study 2 quantitative results is that they reflected condensed programme dosage (and multiple doses over a short interval rather than single doses over a longer interval).

Additionally, while larger sample sizes are often seen as favourable, we argue that demonstrating materials or an intervention does not cause harm should be a moral and ethical priority before any large-scale and/or multi-site trials are progressed. For example, Foulkes and Stringaris (2023) have raised concerns that school-based wellbeing interventions may cause harm, and Hemming et al. (2018) have further argued that excessive cluster sizes have the potential to cause harm via burden and ethical risks to the participants. This has been demonstrated by Deighton et al. (2025), where the wellbeing of over 2,500 children was significantly worse after a 5-week intervention designed to improve mental health and emotional difficulties. To expand, 9 months after the intervention, those children assigned to the intervention (vs. standard practice) demonstrated increased emotional difficulties. Thus, with child populations, which, according to all ethical assessment boards, are classed as vulnerable, we argue that smaller-scale trials should not be seen as a limitation, but are advisable and the way forward until it has been well-established that content does not cause harm.

A more valid limitation of this research is that no longer-term follow-up data were collected. Thus, the longer-lasting benefits of WoBoB are unknown. However, in adapting WoBoB for even younger children aged 9–10, our current unpublished research does appear to demonstrate that effects are maintained at a 3-month follow-up (i.e., Hurst et al., in prep). That said, including follow-up time points at even longer intervals is essential to comprehensively explore effect maintenance and longevity.

A final point of consideration is that rigorous trial design often necessitates the use of language that assigns participants to the ‘intervention’ group. However, WoBoB is not a mental health intervention; thus, where possible, we have used the terminology ‘programme’. Notably, WoBoB consists of good quality SEL materials that should be available to all. Previously, Maratos (2025; see also Montagne et al. 2026) has suggested that the language of scientific trials, in particular, the use of the terms ‘intervention group’ and ‘control group’, may lead to the conflation of good quality SEL with mental health interventions. In the high-quality trial of WoBoB, this limitation of scientific language is important to recognise. Subsequently, it led to our use of the term ‘experimental group’ to ensure that WoBoB, which is universal SEL, is not wrongly classified as a mental health ‘intervention’.

## 6 Conclusion

To conclude, the objectives of the present research were to explore whether the effects of WoBoB: i) extend to younger children and ii) are affected by programme dosage. Study 1 demonstrated that the effects of WoBoB do extend to younger children. Thus, WoBoB is an effective universal (i.e., whole-class) compassion-based SEL programme that positively impacts emotion regulation abilities, compassion, prosocial behaviours, classroom climate, and anxiety. Given that in the UK alone, total annual societal costs per anxious child were £4,040 in 2021 (Pollard et al., 2023), effective strength-based SEL materials, such as WoBoB, could help stem the current unfolding mental health crisis (see also United Nations SDG 3, 2025). However, we caveat this with the findings of Study 2. In Study 2, quantitative data returned null results suggesting that the effects of WoBoB on indices of self-wellbeing (such as anxiety, self-compassion, and general wellbeing) are negated when delivery is compacted. As such, we argue that certain elements of SEL, such as emotion regulation and self-compassion, require time to embed, with opportunities to consolidate learning and practice the introduced skills/practices being a necessary requisite. However, as the qualitative data from Study 2 revealed compacted delivery resulted in a positive classroom climate and changes in prosocial behaviour, this does demonstrate that some effects of WoBoB are maintained with reduced programme dosage. Finally, considering the potential for interventions and SEL materials to cause harm, we suggest that small-scale trials are best practice until rigorous testing demonstrates consistent benefits of an intervention over harm.

## Data Availability

On publication of this article, the quantitative datasets analyzed for this study will be made available on the UDORA repository (https://derby-repository.worktribe.com/).
