# Supplementary material for: Compassionate mind training as school-based social and emotional learning: extension to younger pupils and effects of programme dosage
Source: Front Psychol. 2026 Sep 22;17:1861788. doi: 10.3389/fpsyg.2026.1861788 (PMC13638361; doi:10.3389/fpsyg.2026.1861788)
Supplement: Supplementary file 1 [file Supplementary_file_1.pdf]

Supplementary Materials

Supplementary Table 1: CMT-Pupils WoBoB Lesson content

Lesson 1 focused on ground-rules for the lesson series and an overview of the three emotion systems of the mind/body. The pupil objectives for this lesson included establishing ground rules for the 6 lessons, being able to name the three systems of the mind, being able to give examples of situations or things that can be tied to each system and, between now and the next lesson, identifying which systems they (i.e., each child/pupil) have been in. The latter being referred to as 'The Weekly Challenge' (and not Homework).
Lesson 2 focussed on the drive system. From Lesson 2 onwards, each Lesson began with the Three systems 'Check-in' activity. This is a reflection on the three emotions systems model and discussions as to which system/s each pupil has been in since the last lesson. The pupil objectives for Lesson 2 included: Pupils being able to give examples of when they have been in threat, drive or soothing. Pupils gaining a deeper understanding of drive, including what is going on in both the brain and body when we are in drive. For pupils to be able to recognise when they need to regulate drive (and/or 'over' drive) and, finally, understand practices (and coping techniques) they can engage with to lower drive emotions. Accordingly, in Lesson 2, regal walking and calm imagery were introduced as relevant practices. Following this lesson, the weekly challenge for the Year 6 pupils was to practice calming imagery and/or regal walking when they recognised they needed to regulate their drive emotions. Given Lesson 2 and 3 were progressed in one 'dose' for the Year 7 pupils, please refer to Lesson 3 for the Year 7 pupils Weekly Challenge.
Lesson 3 moved focus to the threat system. Following the check-in activity, core objectives included allowing pupils a deeper understanding of threat, including what is happening in both the brain and the body when we are in threat. For the pupils to learn to recognise when threat is useful and when it is not, as well as understand what might prompt threat in themselves. For the pupils to understand practices and coping techniques they can engage with to move away from threat, over-threat and/or drive emotions*. Accordingly, in Lesson 3, several different breathing techniques were introduced (including soothing and finger breathing), adding to the children's growing toolkit of emotion-regulation techniques. For all children the Weekly Challenge was to practice calming imagery, regal walking and/or breathing regulation techniques when they recognised they needed to regulate drive or threat emotions.

*Note that in application of accurate human physiology, key curriculum content relates to educating children that the sympathetic nervous system is associated with fight, flight and fun responses (cf. fight and flight).
Lesson 4 introduced children to the concept of compassion, compassion-based practices/imagery and the three flows of compassion. Following the check-in activity, main objectives for pupils during this lesson were for pupils to develop a compassionate image, engage in a compassionate imagery practice using this image, and for pupils to gain an understanding of compassion and its three flows. To begin engendering development of the three flows, encouraging compassion towards others was this week's weekly challenge for the Year 6 pupils. Given Lesson 4 and 5 were progressed in one 'dose' for the Year 7 pupils, please refer to Lesson 5 for the Year 7 pupils Weekly Challenge.
Lesson 5 enabled continued consolidation of the three systems model and continued exploration of compassion, by exploring soothing in more depth. Following the check-in activity, main objectives included enabling pupils deeper understanding of soothing including its prompts, thoughts and bodily responses. Enabling pupils to understand the physiology of soothing as compared to threat and drive, through accurate physiology education (i.e., the role of the parasympathetic nervous system). Enabling pupils' experiential practice of soothing through further exploration of compassionate imagery. Accordingly, for the Year 6 pupils, the Weekly Challenge set was to practice compassion for self, whereas for the Year 7 pupils it was to practice both compassion for others and compassion for self.
The purpose of Lesson 6 was continued exploration of compassion, its three flows, and to provide pupils with the skills/practices they need for good emotional regulation and good interpersonal relationships going forward. Following one last check-in activity, objectives included: allowing pupils self-reflection of their emerging emotion regulation skills (via the weekly 'check-in' and 'systems balanced' activities). Enabling continued exploration of compassion and its flows by introduction of a further compassionate practice. Allowing pupils to develop their flows of compassion skills set by an experiential compassionate compliments' activity (involving drive emotions) and, finally, enabling children to recognise that they are becoming wise compassionate individuals, both for themselves and others. For all Year 6 and Year 7 pupils the final challenge set was for them to continue from this point forward practicing compassion for others, for self and from others.

Open-Ended Questions Posed at Time Point Two:

Question 1: Do you think the wellbeing lessons have had any effect on how you feel or on your well-being? For example, has it helped you to understand your emotions? Or understand the emotions of others (e.g. your classmates, friends, family etc.)? You can use the space below to write about what you've learnt that you think is important:

Question 2: Do you think the wellbeing lessons have had any effect on your behaviour? For example, have you tried to use any of the things introduced (e.g. regal walking, calm place imagery, soothing or hand breathing, compassionate imagery)? Or has knowing more about emotions affected how you behave? You can use the space below to tell us about this. You might even be able to think about a specific situation when what you've done in the wellbeing lessons has been really helpful:

Question 3: Do you think the wellbeing lessons have had any effect on your class? For instance, have you noted any changes in how some children in your class behave, or how you behave with some children in your class? Again, you might be able to think about a specific example or you might just have noticed some general things. You can use the space below to tell us anything you think is important about how the lessons have affected your class:

Question 4: We'd also like to ask you what you enjoyed best about the wellbeing lessons, or if there is anything you'd have liked more of? Or was there anything that you did not like? Or is there anything you want to tell us that was not covered in the above questions? Use this space to tell us:

Thematic Analyses Additional Information:

The 11 codes generated by CH on initial review and re-reviews of the Year 6 open question data:

1.Insight into own emotions

2. Understanding relationships

3. Use of Compassion to self and others

4. Help to Cope with difficult emotions

5. Realisation they are not alone

6. Use of techniques

7. Improved relationships with others

8.Techniques change behaviour

9. Class behaviour

10.Enjoyment

11. Positive impact

The 11 codes generated by JH on initial review and re-reviews of the Year 7 open question data:

1. Normalising emotions and self-acceptance
2. Increased emotional understanding of self and others
3. Positive impact on feelings and wellbeing
4. Limited engagement or perceived impact
5. Use of compassion-based techniques
6. Emotional or behavioural changes reported
7. Specific positive impacts at class level
8. General positive impacts on class atmosphere
9. Specific lesson elements or techniques enjoyed
10. General enjoyment of the lessons
11. What pupils wanted more of
